# The Effects of Gene Duplication Modes on the Evolution of Regulatory Divergence in Wild and Cultivated Soybean

**DOI:** 10.3389/fgene.2020.601003

**Published:** 2020-12-08

**Authors:** Na Zhao, Xiaoyang Ding, Taotao Lian, Meng Wang, Yan Tong, Di Liang, Qi An, Siwen Sun, Scott A. Jackson, Bao Liu, Chunming Xu

**Affiliations:** ^1^Department of Agronomy, Jilin Agricultural University, Changchun, China; ^2^Key Laboratory of Molecular Epigenetics of Ministry of Education (MOE), Northeast Normal University, Changchun, China; ^3^Soybean Research Institute, Jilin Academy of Agricultural Sciences, Changchun, China; ^4^Center for Applied Genetic Technologies, University of Georgia, Athens, GA, United States

**Keywords:** soybean, hybrid, regulatory divergence, duplicate gene, *Glycine max*, *Glycine soja*

## Abstract

Regulatory changes include divergence in both *cis*-elements and *trans*-factors, which play roles in organismal evolution. Whole genome duplications (WGD) followed by diploidization are a recurrent feature in the evolutionary history of angiosperms. Prior studies have shown that duplicated genes have different evolutionary fates due to variable selection constraints and results in genomic compositions with hallmarks of paleopolyploidy. The recent sequential WGDs and post-WGD evolution in the common ancestor of cultivated soybean (*Glycine max*) and wild soybean (*Glycine soja*), together with other models of gene duplication, have resulted in a highly duplicated genome. In this study, we investigated the transcriptional changes in *G. soja* and *G. max*. We identified a sizable proportion of interspecific differentially expressed genes (DEGs) and found parental expression level dominance of *G. max* in their F1 hybrids. By classifying genes into different regulatory divergence types, we found the *trans*-regulatory changes played a predominant role in transcriptional divergence between wild and cultivated soybean. The same gene ontology (GO) and protein family (Pfam) terms were found to be over-represented in DEGs and genes of *cis*-only between JY47 and GS, suggesting the substantial contribution of *cis*-regulatory divergences to the evolution of wild and cultivated soybeans. By further dissecting genes into five different duplication modes, we found genes in different duplication modes tend to accumulate different types of regulatory differences. A relatively higher proportion of *cis*-only regulatory divergences was detected in singleton, dispersed, proximal, and tandem duplicates than WGD duplicates and genome-wide level, which is in line with the prediction of gene balance hypothesis for the differential fates of duplicated genes post-WGD. The numbers of *cis*-only and *trans*-only regulated genes were similar for singletons, whereas there were more genes of *trans*-only than *cis*-only in the rest duplication types, especially in WGD in which there were two times more *trans*-only genes than that in *cis*-only type. Tandem duplicates showed the highest proportion of *trans*-only genes probably due to some special features of this class. In summary, our results demonstrate that genes in different duplication modes have different fates in transcriptional evolution underpinned by *cis*- or *trans*-regulatory divergences in soybean and likely in other paleopolyploid higher organisms.

## Introduction

Cultivated soybean (*Glycine max* L. Merr.) is believed to be domesticated from wild soybean (*Glycine soja Sieb*. and *Zucc*.) in East Asia 6,000–9,000 years ago (Kim et al., 2012). However, recent genomic studies suggested that soybean domestication was a complex process involving introgressions between wild and domesticated soybeans (Kim et al., 2010; Li et al., 2014; Wang et al., 2019). Although the origin and domestication of soybean are still under debate, the two species have accumulated enormous genetic and phenotypic changes since their divergence (Gong, 2020). Nevertheless, *G. max* and *G. soja* can be hybridized to form fertile offspring with mostly normal meiotic chromosome pairing. Phenotypic differences between *G. max* and *G. soja* can arise from functional divergence of gene products as well as regulatory divergence of their expression. The evolution in gene products has historically received more attention because they can be easily detected. With the development of new technologies, methods for identifying the genetic changes that underlie expression changes have been developed (Wittkopp et al., 2004; McManus et al., 2010). Transcriptional regulation includes two major components: *cis*-acting elements (i.e., promoters, enhancers, and silencers) and *trans*-acting factors (i.e., transcription factors and non-coding regulatory RNAs). Gene expression is controlled by biochemical interactions between *cis*-acting elements and *trans*-acting factors. Regulatory divergence, including both *cis*- and *trans*-acting changes, can be inferred through comparing differences in gene expression between two genotypes to differences in allelic expression in their F1 hybrids (Wittkopp et al., 2008). Previous studies showed that *trans*-regulatory divergence often make larger contributions to gene expression differences than *cis*-regulatory divergence within species, whereas *cis*-regulatory divergence makes either similar or greater contributions to gene expression divergence between species (Zhuang and Adams, 2007; Wittkopp et al., 2008; Emerson et al., 2010; Goncalves et al., 2012; Lemmon et al., 2014; Xu et al., 2014; Guerrero et al., 2016; Wu et al., 2016). *Cis*-regulatory changes preferentially accumulate over time which fits the theory that *trans*-regulatory changes are selected against by purifying selection and many *cis*-regulatory changes are selected for by positive selection (Prud’homme et al., 2007; Emerson et al., 2010; Coolon et al., 2014). Domesticated plants have experienced unique evolutionary bottlenecks which may lead to differences in the relative contributions of *cis*- and *trans*-regulatory divergence relative to undomesticated taxa (Lemmon et al., 2014).

Whole genome duplications (WGD) or polyploidization are prevalent and recurring throughout the evolutionary histories of all flowering plants (Jiao et al., 2011; Wei and Ge, 2011). Two ancestral WGD events occurred in the common ancestor of seed plants and the common ancestor of angiosperms, respectively (Jiao et al., 2011). The majority of genes duplicated by WGD will return to a single copy over evolutionary time, whereas some duplicated genes will be retained. The fates of duplicated genes following WGD have attracted much interest. Several models have been proposed to explain the loss or retention of duplicated genes. The neofunctionalization and sub-functionalization hypotheses predict that duplicated copies evolve neutrally (Innan and Kondrashov, 2010) and are retained by acquiring new function or reciprocal loss-of-function mutations (He and Zhang, 2005). Another widely accepted hypothesis is the gene balance hypothesis that states the stoichiometry of members of multisubunit complexes affects the function of the whole due to the kinetics and mode of assembly (Birchler and Veitia, 2010). The gene balance hypothesis predicts that all gene duplicates are not retained equally and that loss of dosage-sensitive WGD genes in an interacting balance relationship with others will be selected against in post-WGD evolutionary processes (Birchler and Veitia, 2007, 2010). This has been supported with evidence that WGD-derived duplicated genes are enriched in signal transduction components and transcription factors in multiple plant species. Meanwhile, these functional categories were found to be under-represented in genes duplicated by small-scale duplications e.g., tandem duplication (Blanc and Wolfe, 2004; Maere et al., 2005; Chapman et al., 2006; Xu et al., 2018). Since genes in different duplication modes are the result of and/or under different selection pressures, it is interesting to investigate the relationship of gene duplication mode and types of regulatory divergence.

Besides the two ancient WGDs, *G. max* and *G. soja* experienced two additional sequential WGD events; one occurred about 59 MYA in the common ancestor of legumes and the other about 8–13 MYA in the *Glycine* lineage (Schmutz et al., 2010; Chen et al., 2020). More than 75% of genes in the paleopolyploid soybean genome are multiple copies, and most of these resulted from the WGD events (Schmutz et al., 2010). Recent studies of duplicated genes in soybean showed that genes in different duplication modes have different expressions and gene body DNA methylation profiles (Xu et al., 2018). The functional classification and expression divergence of WGD genes supported different hypotheses of duplicate gene evolution (Xu et al., 2018). The WGD genes in soybean were found to be enriched in *Glycine* transcription factors and transcription regulation functions, which fits the gene balance hypothesis (Xu et al., 2018) and indicates variable constrains on the evolution of genes derived from different duplication modes. In this study, we investigated the transcriptional changes and regulatory divergences as well as their functional preference and relationship with duplication modes in *G. max* and *G. soja*. We reveal the effects of gene duplication modes on the evolution of gene expression and regulation in wild and cultivated soybean.

## Materials and Methods

### Growth Condition, RNA Extraction, and Sequencing

Jiyu47 (JY47) is a soybean elite cultivar which is mainly planted in northeast China. The wild soybean GS was collected from middle China. The hybrid between wild and cultivated soybean was created using JY47 and GS as paternal and maternal parents respectively. The seeds of the three genotypes were planted into soil and grown in a growth chamber under 18-h light and 6-h dark cycles. The temperatures were 25 and 22°C in day and night, respectively. The plants were grown until the first trifoliate was fully developed; then, the second trifoliate leaf was harvested and frozen in liquid nitrogen. For each genotype, three individuals were harvested and stored separately. RNA was extracted for each individual plant using the Trizol method according to the manufacture’s instruction. The total RNA samples were sent to a sequencing company for library construction and sequencing. The sequencing platform was Novaseq 6000. The raw reads were cleaned to remove adapter contamination, low quality reads, and reads with more than 5% N bases. At least 5 Gb of clean bases were produced for each sample.

### RNA-seq Data Processing, Mapping, and Identifying Differentially Expressed Genes

Equal-amount reads from both parental samples were mixed and served as the *in silico* hybrid. Three *in silico* hybrid replicates were created using different parental samples. Then, RNA-seq data were mapped to cultivated soybean reference genome (Williams 82, version: a2v1) using STAR (version 2.7.0d) with settings to report the alignments of uniquely mapped reads (Dobin et al., 2013). Gene expression data were filtered, and genes whose average read counts were bigger than 10 and less than 1,000 were kept. Gene expression levels between genotypes were normalized and compared using DESeq2 with default setting (Wald test) (Love et al., 2014). The differentially expressed genes (DEGs) were identified using a cutoff of FDR adjusted *p*-value < 0.05. The same processes were conducted using wild soybean genome (GCF_004193785.1) as the reference to examine the impacts of mapping preference on the DEG analysis. A detailed description of command and parameters can be found in the supplementary notes.

### DNA Sequencing Data Processing and SNP Calling

The raw DNA sequencing data were filtered to remove adapter contamination, low-quality reads, and reads with more than 5% N bases, then trimmed using “Trimmomatic-0.39” with the parameter “LEADING:5 TRAILING:5 MINLEN:75” (Bolger et al., 2014). Clean reads of the two parental genotypes were mapped against the cultivated reference genome using BWA with default settings (Li and Durbin, 2009). Variants were called using the HaplotypeCaller tool, then both parental genotypes were jointly genotyped using the GenotypeGVCFs tool in GATK (version 4.1.3.0). The raw variants were filtered using VariantFilteration with a setting of “QD < 2.0, QUAL < 30.0, SOR > 3.0, FS > 60.0, MQ < 40.0, MQRankSum < −12.5, and ReadPosRankSum < −8.0.” Then, only bi-allelic SNPs with genotype quality >20 and sample depth >5 were kept. Equal amounts of DNA sequencing reads were mixed and mapped to the reference genome. A detailed description of commands and parameters can be found in the Supplementary Materials.

### Calculating Allelic Expression

The BAM files generated from mapping F1 and *in silico* hybrid RNA-seq data and mixed DNA data were used for allelic analysis. Allelic read counts were calculated at each SNP site using ASEReadCounter tool in GATK. The mapped RNA or DNA reads covering these sites were assigned to JY47 or GS based on the SNPs. SNPs were filtered to remove sites with biased parental DNA read counts (binomial test *p*-value < 0.05 for 1:1 ratio) in the mixed DNA sample. Genes with less than two SNPs between parental genotypes were excluded in further analysis. For each gene, the allele specific expression was calculated by summing the number of JY47 reads or GS reads in the body region. A detailed description of commands and parameters can be found in the Supplementary Materials.

### Assignment of Regulatory Divergence Types and Duplication Modes

The regulatory divergence types were assigned using the method described in McManus et al. (2010). Briefly, the relative allelic expression of every gene was tested in F1 hybrid (named H comparison) and *in silico* hybrid (named P comparison) using binomial test against the null hypothesis of 1:1 respectively, and compared between F1 and *in silico* hybrid (named T comparison) using Fisher’s exact test. The difference was classified as significant in any comparison with the FDR adjusted *p*-value < 0.05. For the relative allelic expression of a gene, the significance in P comparison was considered evidence of parental expression divergence. The expression difference in F1 hybrid (significant in H comparison) was considered evidence of *cis-*regulatory divergence. The parental expression divergence was considered due to *trans*-regulatory changes if the allelic expression was not different in the F1 hybrid (no significance in H comparison) and the ratios of allelic expression were different between the parental mix (*in silico* hybrid) and F1 hybrid (significant in T comparison). The regulatory divergence types were further classified into seven types using the following criteria: *cis*-only: significant in comparison P and H but not significant in T. *trans*-only: significant in comparison P and T, but not H. *cis* + *trans*: significant in comparison P, H, and T, moreover, the log2-transformed allelic expression ratio has the same sign in F1 and *in silico* hybrid. In the *cis* + *trans* type, the *cis*- and *trans-regulatory* divergences favor expression of the same allele. *cis*^∗^*trans*: significant in comparison P, H, and T, besides, the log2-transformed allelic expression ratio has the opposite sign in F1 and *in silico* hybrid. In the *cis*^∗^*trans* type, the *cis*- and *trans*-regulatory divergences favor expression of the opposite alleles. Compensatory: significant in comparison H and T, but not in P. In the compensatory type, the *cis*- and *trans*-regulatory divergences compensate each other. Conserved: no significance in any of the three comparisons. Ambiguous: all other patterns. Genes were classified into five duplication modes using the method described in Xu et al., 2018. The protein sequences of all genes were aligned to each other using blastp program, then the gene duplication modes were assigned using MCScanX (Wang et al., 2012). Genes in singleton mode had no hits in the all-to-all blastp search. Genes in dispersed mode are dispersed paralogs interrupted by many genes on the same chromosome or non-collinear on different chromosome. Genes in proximal mode are paralogs interrupted by fewer than 20 genes. Genes in tandem mode are clusters of consecutive tandem duplicates. Genes in WGD mode are paralogs in collinear chromosome regions. The WGD genes were further classified into old and young ages based on the distribution of *K*_s_ values (Xu et al., 2018). Briefly, the *K*_s_ values between WGD duplicates were calculated using “add_ka_and_ks_to_collinearity.pl” in MCScanX, and the average *K*_s_ value for each collinear block was calculated. The collinear blocks were then clustered into three groups using a *k*-means method (*k* = 3) in R; then genes were classified as young duplicates if present only in the cluster with least mean *K*_s_ value. The other genes were classified as old duplicates because they were found in at least one old cluster.

### GO and Pfam Enrichment Analysis

DEGs between genotypes and genes assigned into different regulatory divergence types were used for functional enrichment analysis. gene ontology (GO) or protein family (Pfam) terms containing less than five expressed genes were removed from further analysis. A one-tail hypergeometric test was used to test whether a GO or Pfam term was over-represented in DEGs or genes of different regulatory types. The raw *p*-values were adjusted using the FDR method, and only terms whose adjusted *p*-value less than 0.05 were classified as significantly over-represented.

## Results

### Gene Expression Changes in Cultivated and Wild Soybean, and Their Hybrid F1

The cultivated soybean JY47 (*G. max*) and the wild soybean GS (*G. soja*) are dramatically different in morphology, while F1 hybrids between them show intermediate phenotype for many traits, such as plant height and leaf size (Supplementary Figure 1). RNA-seq reads were mapped to the reference genome of cultivated soybean cv. Williams 82 (version a2v1) and the gene expression values were calculated and compared between genotypes. Consistent with morphological differences, 12,677 genes were identified as DEGs between JY47 and GS, which accounted for 43.40% of all expressed genes (29,235) in the leaf tissue. There were nearly equal amounts of up-regulated genes in JY47 (6,321 genes) and GS (6,356 genes) compared with each other. Three mixtures using equal amounts of maternal and paternal data from three pairs of parental individuals were constructed and served as *in silico* “hybrids.” The gene expression values detected in the *in silico* “hybrids” represent additive mid-parental expression levels. In the comparison between F1 hybrids and *in silico* “hybrids,” 493 genes were found to be differentially expressed (non-additive). Interestingly, the down-regulated genes (353 genes) in F1 hybrid were twofold more than the up-regulated genes (140 genes) as compared to *in silico* “hybrids” indicating complicated regulatory interactions in the F1 hybrids. When compared to the two parental genotypes, the F1 hybrids showed more DEGs with GS (5,210) than with JY47 (1,008) (Table 1), indicating the dominant role of regulatory alleles from cultivated soybean. To examine whether the observed parental expression level dominance is due to mapping preference of reads from JY47 to the cultivated reference genome, we performed the same DEGs analysis using a wild soybean reference genome and found the same trend (Supplementary Table 1).

**TABLE 1:** Summary of differentially expressed genes in each comparison between genotypes.

Comparisons	DEGs	Up-regulated^a^	Down-regulated^b^
GS vs. JY47	12,677 (43.4%)	6,356 (21.8%)	6,321 (21.6%)
GS vs. F1	10,048 (34.4%)	4,838 (16.6%)	5,210 (17.8%)
JY47 vs. F1	1,753 (6.0%)	745 (2.5%)	1,008 (3.5%)
F1 vs. Mix*	493 (1.7%)	140 (0.5%)	353 (1.2%)

*^*a*^Number and fraction of DEGs up-regulated in the former genotype. ^*b*^Number and fraction of DEGs down-regulated in the former genotype. ^∗^Mix was constructed by equal amounts of maternal and paternal data and served *in silico* “hybrids.”*

### Regulatory Divergence Between the Wild and Cultivated Soybean Genotypes

**FIGURE 1:**
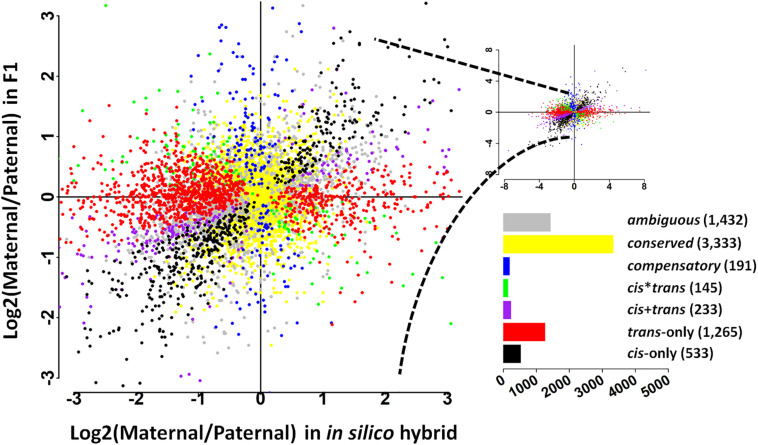
Plot summarizes the relative, allele-specific expression levels in parental (*in silico* hybrid) and the F1 hybrids. Each gene is shown as a point which is color-coded according to the pattern of regulatory divergence. The bar graph depicts the total number of genes in each pattern.

To further address the evolution of expression divergence between the wild and cultivated soybean genotypes, we classified the genes into seven regulatory divergence types based on their allelic expression patterns in the *in silico* “hybrids” and F1 hybrids. In total, 7,132 genes were interrogated, the majority of which were found to be conserved (3,333 genes) or ambiguous (1,432 genes) (Figure 1); 533 genes were diverged in a *cis*-only pattern, while 1,265 were in *trans*-only pattern suggesting *trans*-regulatory changes play a predominant role in the expression divergence between the wild and cultivated soybean genotypes (Figure 1). A relatively lower fraction of genes were found in the other three more complex types (233 in *cis* + *trans*, 145 in *cis*^∗^*trans*, and 191 in compensatory patterns) (Figure 1).

### The Relationship Between Duplication Mode and Regulatory Divergence

To address the relationship between gene duplication modes and type of regulatory divergence, we classified all chromosomal genes into five different categories based on their duplicate states in the reference genome as singleton, dispersed, proximal, tandem, and WGD/large segmental duplication (WGD for short). We calculated the distribution of genes in different regulatory divergence types for each duplication mode. In singletons, we found the same number of genes diverged in *cis-*only and *trans*-only patterns (11% *cis*-only/*trans*-only) but significantly more genes in *trans*-only than *cis*-only divergence type in the other three duplication modes (chi-squared test *p*-value < 0.01) (Table 2). The difference in the proportion of genes subject to *cis*-only and *trans*-only patterns was the highest in WGD genes where there were two times more genes in *trans*-only pattern (1,110 genes) than *cis*-only pattern (428 genes) (Table 2). All duplication modes except for WGD mode showed higher proportions *cis*-only genes as compared to genome-wide levels, and differences were statistically significant for singleton and proximal modes (chi-squared test *p*-value < 0.05). Furthermore, the conserved regulatory type accounted for 47.23% of WGD genes, which was the highest, while similar proportions of conserved genes were found in singleton (46.96%) and dispersed (46.56%) genes, whereas proportions in proximal (34.31%) and tandem (37.19%) mode were significantly lower than the genome-wide level (Table 2).

**TABLE 2:** Number and proportion of genes in different regulatory patterns for each duplicate mode.

	*Cis*-only	*Trans*-only	*Cis* + *trans*	Cis*trans	Compensatory	Conserved	Ambiguous
Singleton	22	22	11	2	9	85	30
	(12.15%)	(12.15%)	(6.08%)	(1.10%)	(4.97%)	(46.96%)	(16.57%)
Dispersed	45	76	14	7	17	230	105
	(9.11%)	(15.38%)	(2.83%)	(1.42%)	(3.44%)	(46.56%)	(21.26%)
Proximal	15	18	11	2	4	35	17
	(14.71%)	(17.65%)	(10.78%)	(1.96%)	(3.92%)	(34.31%)	(16.67%)
Tandem	22	44	11	6	6	74	36
	(11.06%)	(22.11%)	(5.53%)	(3.02%)	(3.02%)	(37.19%)	(18.09%)
WGD	428	1100	185	128	155	2,898	1,242
	(6.98%)	(17.93%)	(3.01%)	(2.09%)	(2.53%)	(47.23%)	(20.24%)
Total	532	1,260	232	145	191	3,322	1,430
	(7.48%)	(17.72%)	(3.26%)	(2.04%)	(2.69%)	(46.71%)	(20.11%)

### Functional Enrichment of DEGs and Genes in Different Regulatory Divergence Types

**FIGURE 2:**
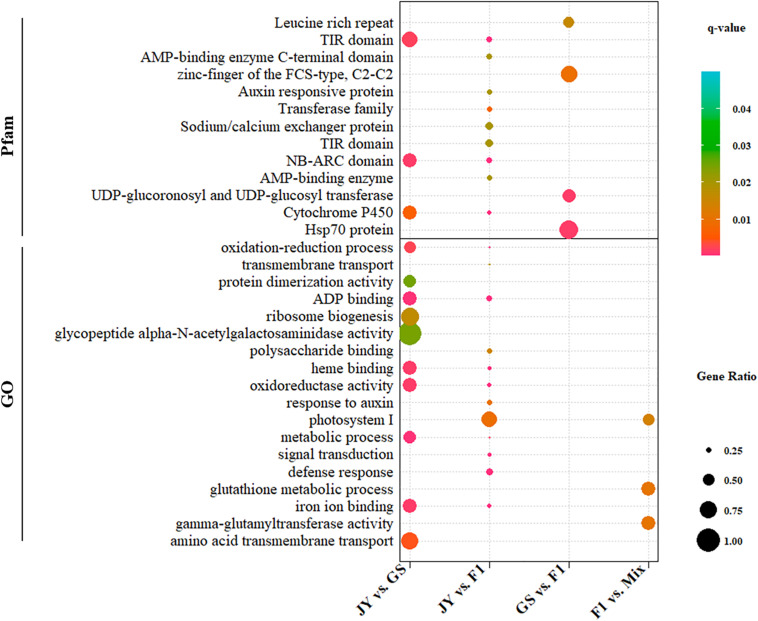
GO and Pfam enrichment of DEGs between genotypes. GO (Gene Ontology) (lower) or Pfam (upper) terms significantly (*q*-value < 0.05) that were over-represented in DEGs are shown. The dot size and color correspond to proportions of DEGs and the *q*-values.

We performed GO and Pfam enrichment analysis for DEGs and genes in different regulatory divergence types. DEGs between cultivated and wild soybean showed significant enrichment in ADP-binding (GO:0043531), oxidation-reduction related terms (GO:0016705, GO:0055114) and other GO terms, totaling to five GO terms (*q*-value < 0.05) (Figure 2 and Supplementary Material). Three GO terms, gamma-glutamyltransferase activity, glutathione metabolic process, and photosystem I, were over-represented in non-additive genes (Figure 2 and Supplementary Material). Three protein domains, NB-ARC domain, TIR domain, and cytochrome P450 were over-represented in DEGs between the two parental genotypes; however, no protein domain was over-represented in the non-additive genes (Figure 2 and Supplementary Material).

For genes in different regulatory divergence types, ADP binding GO term was over-represented in genes of *cis*-only type, whereas no GO terms were over-represented in other regulatory types except protein binding (GO:0005515) in conserved pattern (Supplementary Material). Different protein families (Pfam domain) were over-represented in genes of *cis*-only and *trans*-only patterns. NB-ARC domain and TIR domain were over-represented in genes of *cis*-only pattern, while the response regulator receiver domain and Hsp70 protein domain were over-represented in *trans*-only pattern genes (Supplementary Material). No over-represented domain was found in the rest of the regulatory divergence patterns. The top over-represented GO term (ADP binding) and Pfam terms (NB-ARC domain and TIR domain) were the same in DEGs between GS and JY and genes of *cis*-only divergence.

## Discussion

In line with the differences in plant morphology, a large number of genes were found to be differentially expressed between JY47 and GS, indicating that domestication and subsequent evolution/improvement have dramatically shaped the transcriptomes of *G. max* and *G. soja*. Commonality of the top over-represented GO and Pfam terms in DEGs and genes subject to *cis*-only regulatory divergence between JY47 and GS (Supplementary Material) suggests the substantial contribution of *cis*-regulatory divergence in the evolution and diversification of wild and cultivated soybeans. A few studies have shown that gene expression changes played a role in the domestication and improvement of soybean (Dai et al., 2018; Zhang et al., 2019; Miao et al., 2020). One example is the *GmCYP78A* gene family of which there are three members: two, *GmCYP78A70* (Glyma.01G061100) and *GmCYP78A57* (Glyma.02G119600), were derived from a single ancestor during the latest WGD ∼13 Mya, and the third copy *GmCYP78A72* (Glyma.19G240800) was duplicated from *GmCYP78A57* (Dai et al., 2018). These genes show expression divergence among tissues and positive correlation with leaf size and seed weight in different cultivars; furthermore, population genetic results indicate two underwent intense selection during soybean domestication and/or improvement (Dai et al., 2018). In our study, *GmCYP78A70* and *GmCYP78A57* showed detectable expression in leaf (Supplementary Figure 2) and the expression *GmCYP78A70* in cultivated soybean was statistically higher than in wild soybean consistent with the previous study. Genome-wide expression levels in the hybrid were biased toward the cultivated soybean JY47, indicating parental expression level dominance (Table 1 and Supplementary Table 1). A similar phenomenon has been found in cotton (Flagel et al., 2008) which was shown to result from the up- or down-regulation of gene copy (allele/homeolog) from the non-dominant parent (Yoo et al., 2013). Expression level dominance can be caused by *trans*-regulatory interactions, which accords with our findings of a large proportion of genes subject to *trans*-regulatory divergence.

The relative contribution of *cis*- and *trans*-regulatory divergence in evolution has been broadly studied. *Trans*-regulatory divergence has been found to play a dominant role in the regulatory divergence within species, while *cis*-regulatory divergence makes either similar or greater contribution to gene expression divergence between species (Zhuang and Adams, 2007; Wittkopp et al., 2008; Emerson et al., 2010; Goncalves et al., 2012; Lemmon et al., 2014; Xu et al., 2014; Guerrero et al., 2016; Wu et al., 2016), which fits the prediction of different types of selection acting on the two types of regulatory divergences (Prud’homme et al., 2007; Emerson et al., 2010; Coolon et al., 2014). Besides the complicated divergence and domestication history, *G. max* and *G. soja* have a highly duplicated genome due to the two recent WGDs in their common ancestor occurred about 13 MYA and 59 MYA (Schmutz et al., 2010). The gene balance hypothesis predicts that all gene duplicates are not equally retained following a WGD (Edger and Pires, 2009; Birchler, 2019); therefore, genes resulting from different duplication modes in the soybean genome have experienced different selection constraints. Prior studies have shown there are abundant genes in different duplication modes, but >60% of genes remain collinear in the soybean genome (Xu et al., 2018). The WGD genes in soybean were found to be enriched in transcription factors and transcription regulation functions, which is in line with the gene balance hypothesis. In soybean, different duplication modes are distinct from each other in DNA methylation and expression profiles as well as enriched functional categories (Xu et al., 2018), suggesting varied constraints on the evolution of genes in different modes.

In this study, we revealed the effects of duplication mode on the evolution of regulatory divergence between wild and cultivated soybean. We found that genes from different duplication modes tended to accumulate different types of regulatory divergence. Relative higher proportions of *cis*-only regulatory divergence were detected in singleton, dispersed, proximal, and tandem modes than in genes from a WGD and genome-wide levels (Table 2), consistent with the prediction of gene balance hypothesis that genes in these duplication modes are less involved in regulatory networks (Edger and Pires, 2009). However, at genome-wide scale, *trans*-regulatory changes were found to play a predominant role in the expression divergence between *G. soja* and *G. max* (Figure 1). We found that as majority constituents to the soybean genome, WGD genes are more likely to be affected by *trans*-regulatory changes than by *cis*-regulatory changes, leading to the observed more *trans*-regulatory changes in genome-wide scale (Table 2). Some WGD duplicates may have conserved regulatory regions following whole genome duplications. These paralogs can be regulated by the same *trans-*acting factors which can lead to amplified effects of *trans-*regulatory changes in these genes. Furthermore, the retained WGD genes are more likely involved in regulatory network according to gene balance hypothesis. Transcription factors are usually dosage-sensitive and preferentially retained following WGDs due to dosage constraint, which has also been supported in a previous study in soybean (Xu et al., 2018). In this study, we observed a high proportion of WGD genes in *trans-*only regulatory type. Genes affected by *trans-*regulatory divergence were more likely to be the targets of transcription factors. Here, our results suggest that not only the transcription factors but also many of their targets have been retained in the collinear blocks in the soybean genome which have experienced transcriptional divergence. However, it is still not clear how the diverged *trans*-acting factors are released from purifying selections and gene balance constrains. The proportion of conserved genes was highest in WGD mode suggests they are under stronger purifying selection than genes in other duplication modes. The expression coordinates of retained WGD paralogs were decreased and transcriptional divergence increased over time in soybean (Xu et al., 2018). Expression divergence indicating subfunctionalization and/or neofunctionalization contributes to the maintenance of most duplicated regulatory genes in *Arabidopsis* after each round of duplication (Duarte et al., 2006). A recent study in *Paramecium* and yeast revealed that WGD genes were retained due to dosage constraint followed by divergence in expression level and eventual deterministic gene loss through dosage subfunctionalization (Gout and Lynch, 2015). Our results revealed the divergence of regulatory network during post-WGD evolution, which is consistent with findings in yeast demonstrating rapid divergence and increase in complexity of networks after polyploidization (Teichmann and Babu, 2004; Gu et al., 2005). Thus, gene/genome duplication plays a key role in network evolution. Together, it is clear that genes in different duplication modes which are under and/or resulting from selection pressures have differential effects on transcriptional evolution due to *cis*- and *trans*-regulatory divergence and that retained WGD genes are prone to *trans*-regulatory divergence. We further classified WGD genes into young and old WGD duplicates based on their Ks values. Most WGD genes (32,993) were young duplicates. A higher proportion of *cis*-only genes (7.11%) but lower proportion of *trans*-only genes (17.59%) were found in young WGD duplicates than in old duplicates (*cis*-only: 5.72%, *trans*-only: 21.14%) (Supplementary Table 2). The proportions of *cis*-only genes in both young and old WGD duplicates were lower than in the other duplicate modes. This is probably due to the large amount of young WGD genes, some of which were less likely to be subject to gene balance constraints and more susceptible to *cis*-regulatory changes than old WGD genes.

Tandem duplicates have the lowest proportion of genes in conserved patterns, suggesting higher divergence rates in these genes. We have shown previously that tandem duplicate genes in the soybean genome are enriched for stress related functions (Xu et al., 2018). Also, there is no evidence implicating that this type of duplicated genes are subject to gene balance constraint (Edger and Pires, 2009). Interestingly, we found the highest proportion of genes due to *trans*-only divergences in the tandem duplicate mode. A recent study also showed that subfunctionalization of expression evolves slowly in tandem duplicates possibly because they are coregulated by shared genomic elements (Lan and Pritchard, 2016). We suggest that coregulation, together with preference of some *trans*-acting factors for tandem duplicates, may have given rise to the observed high *trans*-regulatory divergence in this type of duplicates.

## Data Availability Statement

The datasets presented in this study can be found in online repositories. The names of the repository/repositories and accession number(s) can be found below: https://www.ncbi.nlm.nih.gov/, PRJNA660310 and https://www.ncbi.nlm.nih.gov/, PRJNA660313.

## Author Contributions

CX designed the research. NZ, XD, TL, MW, YT, DL, QA, and SS performed the experiments and analyzed the data. NZ and CX wrote the manuscript. SJ and BL revised the manuscript. All authors read and approved the final manuscript.

## Conflict of Interest

The authors declare that the research was conducted in the absence of any commercial or financial relationships that could be construed as a potential conflict of interest.
