# Supplementary material for: The Effects of Gene Duplication Modes on the Evolution of Regulatory Divergence in Wild and Cultivated Soybean
Source: Front Genet. 2020 Dec 8;11:601003. doi: 10.3389/fgene.2020.601003 (PMC7753205; doi:10.3389/fgene.2020.601003)
Supplement: Supplementary file 2 [file Data_Sheet_2.docx]

Supplementary Material

# Supplementary Data

List of all over-represented GO or Pfam terms in DEGs and each regulatory type.

Supporting Dataset.xlsx

# Supplementary Figures and Tables

## Supplementary Figures


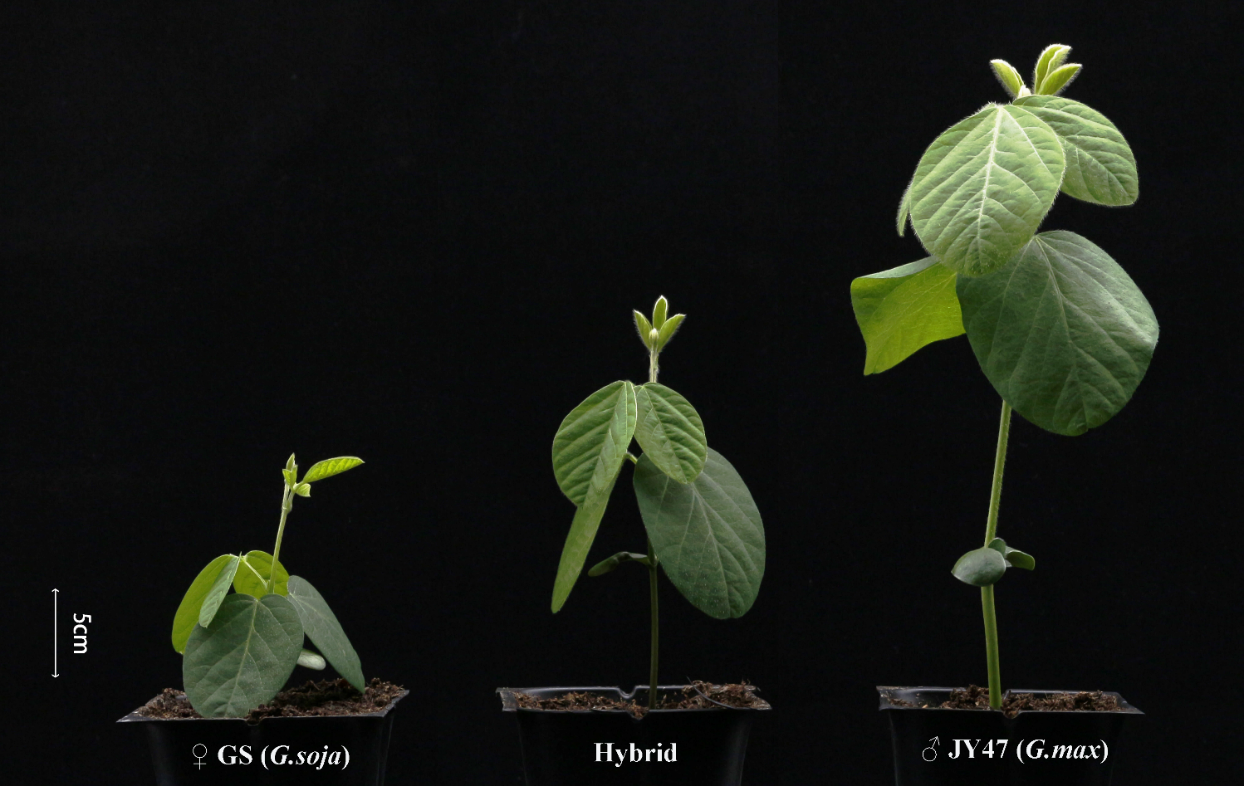
**Supplementary Figure S1.** Phenotypic characteristics of the hybrid and its parents.

**Supplementary Figure S2.** Normalized RNA-seq read counts in JY47 and GS samples.

**
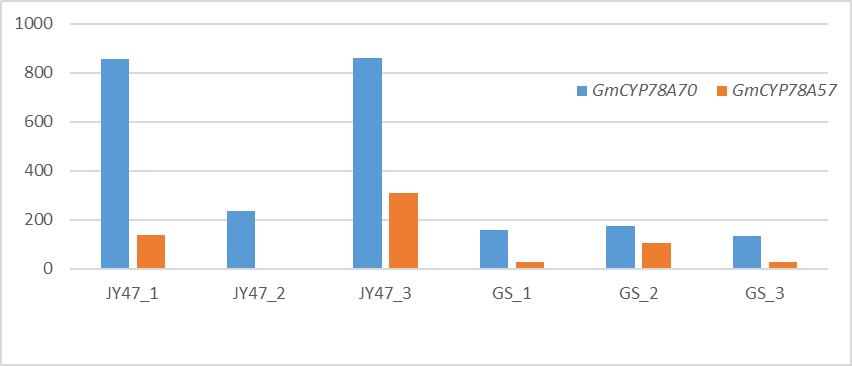
**

**Supplementary** **Table S1. Summary of differentially expressed gens in each comparison between genotypes using the wild soybean reference genome.**

| **Comparisons** | **DEGs** | **Upregulated^a^** | **Downregulated^b^** |
| --- | --- | --- | --- |
| **GS** vs. **JY47** | **12,177** (43.7%) | **6,107** (21.9%) | **6,070** (21.8%) |
| **GS** vs. **F1** | **9,694** (34.8%) | **4,697** (16.9%) | **4,997** (17.9%) |
| **JY47** vs. **F1** | **1,673** (6.0%) | **692** (2.5%) | **981** (3.5%) |
| **F1** vs. **Mix*** | **443** (1.6%) | **119** (0.4%) | **324** (1.2%) |

^a^ Number and fraction of DEGs upregulated in the former genotype.

^b^ Number and fraction of DEGs downregulated in the former genotype.

^*^ Mix was constructed by equal amounts of maternal and paternal data and served *in silico* “hybrids”.

**Supplementary** **Table S2. Number and proportion of genes in different regulatory patterns for young and old WGD genes.**

|  | ***cis*-only** | ***trans*-only** | ***cis*+*trans*** | ***cis***trans*** | **compensatory** | **conserved** | **ambiguous** |
| --- | --- | --- | --- | --- | --- | --- | --- |
| **Young** | 395 | 978 | 164 | 109 | 131 | 2648 | 1134 |
|  | 7.11% | 17.59% | 2.95% | 1.96% | 2.36% | 47.63% | 20.40% |
| **Old** | 33 | 122 | 21 | 19 | 24 | 250 | 108 |
|  | 5.72% | 21.14% | 3.64% | 3.29% | 4.16% | 43.33% | 18.72% |

# Supplementary Notes

Commands and parameters used in SNP calling, allele specific expression and DEG analysis.

**1. Map DNA-seq reads to references and call SNPs**

1.1 Prepare the reference genome sequences

bwa index ./DB/Gmax_275_v2.0.fa

samtools faidx ./DB/Gmax_275_v2.0.fa

java -Xmx64g -jar picard.jar CreateSequenceDictionary REFERENCE=./DB/Gmax_275_v2.0.fa OUTPUT=./DB/Gmax_275_v2.0.dict

########################

1.2 quality filter of sequencing data

##JY47 is used as an example to show the pipeline

mkdir Cleandata

mkdir Log

 java -jar trimmomatic-0.39.jar PE -threads 10 ./Data/JY_DNA_R1.fq.gz ./Data/JY_DNA_R2.fq.gz ./Cleandata/JY_DNA_trimed_R1.fq.gz ./Cleandata/JY_DNA_R1_unpaired.fq.gz ./Cleandata/JY_DNA_trimed_R2.fq.gz ./Cleandata/JY_DNA_R2_unpaired.fq.gz LEADING:5 TRAILING:5 MINLEN:75 &> ./Log/JY_DNA_trimmomatic.log

################################################

1.3 Reads mapping

mkdir BAM

 bwa mem -M -R '@RG\tID:JY_DNA\tSM:JY_DNA\tPL:illumina\tLB:JY_DNA\tPU:JY_DNA' -t 10 ./DB/Gmax_275_v2.0.fa ./Cleandata/JY_DNA_trimed_R1.fq.gz ./Cleandata/JY_DNA_trimed_R2.fq.gz | samtools view -bSh -t ./DB/Gmax_275_v2.0.fai - > ./BAM/JY_DNA.bam

################################################

1.4 Variant calling and genotyping

##JY47 is used as an example to show the pipeline

SampleID="JY"

REF="./DB/Gmax_275_v2.0.fa"

PICARD="/ Program/picard_2.18.27/picard.jar"

GATK="/ Program/gatk-4.1.3.0/gatk-package-4.1.3.0-local.jar"

java -Xmx32g -jar ${GATK} SortSam -I ./BAM/${SampleID}.bam -O ${SampleID}_sorted.bam --SORT_ORDER coordinate --TMP_DIR ./tmp &> ./Log/${SampleID}_picardSortSam.log

java -Xmx32g -jar ${GATK} MarkDuplicates -I ${SampleID}_sorted.bam -O ./${SampleID}.bam --CREATE_INDEX true --VALIDATION_STRINGENCY SILENT -M ./Log/${SampleID}_mkdup.metric --TMP_DIR ./tmp &> ./Log/${SampleID}_picardMarkDuplicates.log

java -Xmx32g -jar ${GATK} HaplotypeCaller -I ./${SampleID}.bam -R ${REF} --min-base-quality-score 30 -ERC GVCF --pcr-indel-model NONE -O ./temp_vcf/${SampleID}.g.vcf.gz &> ./Log/${SampleID}_HC_log.txt

##The same pipeline was used for GS data

################################################

1.5 Joint genotyping

##The raw variant calling results for JY47 and GS were required. A list of chromosome/scaffold names was required.

1.5.1 Joint genotyping for each chromosome

LIST=`zcat ./list_of_chromsome_scaffold_name.txt`

for CHR in $LIST

do

REF="./DB/Gmax_275_v2.0.fa"

PICARD="/ Program/picard_2.18.27/picard.jar"

GATK="/ Program/gatk-4.1.3.0/gatk-package-4.1.3.0-local.jar"

mkdir VCF_chr

mkdir GDB

mkdir GT_tmp_${CHR}

java -Xmx32g -jar ${GATK} GenomicsDBImport --genomicsdb-workspace-path ./GDB/GM_${CHR} -L ${CHR} --tmp-dir=./GT_tmp_${CHR} -V ./temp_vcf/JY47.g.vcf.gz -V ./temp_vcf/GS.g.vcf.gz &> ./Log/GenomicsDBImport_${CHR}.log

java -Xmx32g -jar ${GATK} GenotypeGVCFs -R ${REF} -O ./VCF_chr/GM_${CHR}.g.vcf.gz -V gendb://GDB/GM_${CHR} --tmp-dir=./GT_tmp_${CHR} &> ./Log/GenotypeGVCFs_${CHR}.log

rm -r GT_tmp_${CHR}

done

#######################

1.5.2 Combine chromosomal VCF files into one file

REF="./DB/Gmax_275_v2.0.fa"

GATK="/ Program/gatk-4.1.3.0/gatk-package-4.1.3.0-local.jar"

mkdir VCF

mkdir GT_tmp

LIST=`zcat ./list_of_chromsome_scaffold.txt`

for CHR in $FILES

do

samp+="-I ./VCF_chr/GM_${CHR}.g.vcf.gz ";

done

java -Xmx64g -jar ${GATK} GatherVcfs -R ${REF} -O ./VCF/GM_GATK.g.vcf.gz --CREATE_INDEX true --TMP_DIR=./GT_tmp $samp&> ./Log/GatherVcfs.log

rm -r GT_tmp

################################################

1.6 Select and filter SNPs

REF="./DB/Gmax_275_v2.0.fa"

GATK="/ Program/gatk-4.1.3.0/gatk-package-4.1.3.0-local.jar"

bcftools="/Program/bcftools/bcftools"

java -Xmx32g -jar ${GATK} IndexFeatureFile \

-F ./VCF/GM_GATK.g.vcf.gz

java -Xmx32g -jar ${GATK} SelectVariants \

-R ${REF} \

-V ./VCF/GM_GATK.g.vcf.gz \

-select-type SNP \

-O ./VCF/GM_GATK_snps.vcf.gz &> ./Log/Select_SNP.log

java -Xmx32g -jar ${GATK} VariantFiltration \

-R ${REF} \

-V ./VCF/GM_GATK_snps.vcf.gz \

-filter "QD < 2.0" --filter-name "QD2" \

-filter "QUAL < 30.0" --filter-name "QUAL30" \

-filter "SOR > 3.0" --filter-name "SOR3" \

-filter "FS > 60.0" --filter-name "FS60" \

-filter "MQ < 40.0" --filter-name "MQ40" \

-filter "MQRankSum < -12.5" --filter-name "MQRankSum-12.5" \

-filter "ReadPosRankSum < -8.0" --filter-name "ReadPosRankSum-8" \

-O ./VCF/GM_GATK_snps_filtered.vcf.gz &> ./Log/Filter_SNP.log

##variant quality, depth and bi-allelic SNPs

${bcftools} view --genotype ^het -m2 -M2 -v snps -i'GT!="." & QUAL>20 & MIN(FMT/DP)>5 & MAX(FMT/DP)<50 & MIN(FMT/GQ)>20 & COUNT(GT="RR")>0 & COUNT(GT="AA")>0 & COUNT(GT="het")=0' ./VCF/GM3_GATK_snps_filtered.vcf.gz -Oz -o ./VCF/GM3_biSNP.vcf.gz

${bcftools} index -t ./VCF/GM3_biSNP.vcf.gz

################################################

**2. Map RNA-seq reads to references and count reads per gene using STAR**

2.1 prepare reference and annotation files

PICARD="/ Program/picard_2.18.27/picard.jar"

STAR="/ Program/STAR-2.7.3a/bin/Linux_x86_64/STAR"

gffread ./annotation/Gmax_275_Wm82.a2.v1.gene_exons.gff3 -T -o ./annotation/Gmax_275_Wm82.a2.v1.gene_exons.gtf

samtools faidx DB/Gmax_275_v2.0.fa

java -Xmx64g -jar ${PICARD} CreateSequenceDictionary REFERENCE=./DB/Gmax_275_v2.0.fa OUTPUT=./DB/Gmax_275_v2.0.dict

${STAR} --runMode genomeGenerate --genomeDir ./DB --genomeFastaFiles ./DB/Gmax_275_v2.0.fa --runThreadN 10

################################################

2.2 mapping and counting

##JY47 is used as an example to show the pipeline

mkdir counts

mkdir BAM

mkdir STAR_Log

STAR="/ Program/STAR-2.7.3a/bin/Linux_x86_64/STAR"

SampleID="JY47"

REF="./DB/Gmax_275_v2.0.fa"

R1="./Cleandata/${SampleID}_R1.fq.gz"

R2="./Cleandata/${SampleID}_R2.fq.gz"

Threads=10

####

${STAR} --genomeDir ./DB --readFilesIn ${R1} ${R2} --readFilesCommand zcat --sjdbGTFfile ./annotation/Gmax_275_Wm82.a2.v1.gene_exons.gtf --sjdbGTFtagExonParentGene gene_id --runThreadN ${Threads} --outFilterMultimapNmax 1 --outSAMtype BAM Unsorted

rm Aligned.out.bam

mkdir DB_2pass

${STAR} --runMode genomeGenerate --genomeDir DB_2pass --genomeFastaFiles ${REF} --sjdbFileChrStartEnd ./SJ.out.tab --sjdbOverhang 100 --runThreadN ${Threads}

mkdir 2pass

${STAR} --genomeDir ./DB_2pass --readFilesIn ${R1} ${R2} --readFilesCommand zcat --sjdbGTFfile ./annotation/Gmax_275_Wm82.a2.v1.gene_exons.gtf --sjdbGTFtagExonParentGene gene_id --runThreadN ${Threads} --outFilterMultimapNmax 1 --outSAMmapqUnique 60 --quantMode GeneCounts --outSAMtype BAM Unsorted

mv Aligned.out.bam ./BAM/${SampleID}.bam

mv ReadsPerGene.out.tab ./counts/${SampleID}.counts

mv Log.final.out ./STAR_Log/${SampleID}.Log.final.out

mv Log.out ./STAR_Log/${SampleID}.Log.out

mv SJ.out.tab ./STAR_Log/${SampleID}.SJ.out.tab

mv Log.progress.out ./STAR_Log/${SampleID}.Log.progress.out

## the *.counts files were then merge into one table for further DEG analysis

################################################

**3. Analyze allele specific expression in hybrid and mix**

## the BAM files produced by STAR/BWA and the VCF file of bi-allelic SNPs are required

##F1 is used as an example to show the pipeline.

SampleID="F1"

PICARD="/ Program/picard_2.18.27/picard.jar"

GATK="/ Program/GATK3/GenomeAnalysisTK_3.8.jar"

REF="./DB/Gmax_275_v2.0.fa"

SITES="./VCF/GM_biSNP.vcf"

mkdir ASE_count

OUTPUT="./ASE_count/${SampleID}_ASE_count.csv"

BAM="./BAM/${SampleID}.bam"

java -Xmx16g -jar ${PICARD} SortSam MAX_RECORDS_IN_RAM=1000000 SO=coordinate CREATE_INDEX=true TMP_DIR=tmpdir I=${BAM} O=./${SampleID}_sorted.bam

java -Xmx32g -jar ${GATK} -R ${REF} -T ASEReadCounter -o ${OUTPUT} -I ${SampleID}_sorted.bam -sites ${SITES} -U ALLOW_N_CIGAR_READS -minDepth 0 --minMappingQuality 20 --minBaseQuality 20 --countOverlapReadsType COUNT_FRAGMENTS

## The mapped RNA in F1 and *in silico* hybrid and DNA reads in mixed DNA data assigned to JY47 or GS based on the SNPs using some customized scripts. SNPs were filtered to remove sites with biased parental DNA reads counts (binomial test p-value < 0.05 for 1:1 ratio) in the mixed DNA sample. Genes with less than two SNPs between parental genotypes were excluded in further analysis. For each gene, the allele specific expression was calculated by summing the number of JY47 reads or GS reads in the body region. The relative allelic expression of every gene was tested in F1 hybrid (named H comparison) and in silico hybrid (named P comparison) using binomial test (binom.test in R) against the null hypothesis of 1:1 respectively and compared between F1 and *in silico* hybrid (named T comparison) using Fisher’s exact test (chisq.test in R).

The regulatory divergence types were further classified into 7 types using the following criteria: (1) **cis-only**, significant in comparison P and H but not significant in T. (2) **trans-only**, significant in comparison P and T, but not H. (3) **cis + trans**, significant in comparison P, H and T, moreover, the log2-transformed allelic expression ratio has the same sign in F1 and *in silico* hybrid. (4) **cis*trans**, significant in comparison P, H and T, besides, the log2-transformed allelic expression ratio has the opposite sign in F1 and *in silico* hybrid. (5) **Compensatory**, significant in comparison H and T, but not in P. (6) **Conserved**, no significance in any of the three comparisons. (7) **Ambiguous**, all other patterns. All statistics were perform in R version 3.6.2.

################################################

**4. Identify differentially expressed genes using DESeq2**

##the gene counting results produced by STAR were combine into one file “gene_rawcount.txt” and used in the following analysis in R.

library( "DESeq2" )

dir.create("./DEG")

dir.create("./output")

##read in the gene counting matrix

exp_data.raw <- read.table("./data/gene_rawcount.txt", header = TRUE)

##remove suffix in gene name

exp_data.raw$gene <- gsub(".Wm82.a2.v1", "", as.vector(exp_data.raw$gene))

##remove non-gene information

rm_id <- c("N_unmapped","N_multimapping","N_noFeature","N_ambiguous")

clean_id <- setdiff(exp_data.raw$gene, rm_id)

exp_data <- merge(exp_data.raw, data.frame(gene=clean_id), by ="gene")

## JY: JY47, GS: G.soja, H: F1 hybrid, M: mix/in silico hybrid

sample_list <- c("JY_1", "JY_2", "JY_3", "GS_1", "GS_2", "GS_3", "H_1","H_2","H_3","M_1","M_2","M_3")

count_data_raw <- exp_data[,c(sample_list)]

rownames(count_data_raw) <- exp_data$gene

## to remove genes with too low or too high counts

count_mean <- apply(count_data_raw,1, mean)

count_data <- count_data_raw[count_mean > 10 & count_mean < 1000,]

#############Annotate samples############

gt <- c("JY", "GS", "F1","Mix")

colData <- data.frame(row.names=names(count_data),

gt =rep(gt, each=3))

dds <- DESeqDataSetFromMatrix(

countData=count_data,

colData=colData,

design=~ gt)

####Normalization ########################

dds <- estimateSizeFactors(dds)

norm_data <- counts(dds, normalized=T)

colnames(norm_data) <- paste0(rep(gt,each=3),"_",rep(1:3, length(gt)))

write.csv(data.frame(gene=rownames(norm_data), norm_data),"./output/exp_normalized.csv",

row.names =FALSE,quote = FALSE)

###### compare gene expression between genotypes#######

dds <- DESeq(dds)

resultsNames(dds)

comp_pair <- list( c("JY", "GS"),

c("JY", "F1"),

c("JY", "Mix"),

c("GS", "F1"),

c("Mix", "F1"),

c("GS", "Mix")

)

for (x in 1:length(comp_pair)){

res_sel <- results(dds, contrast=c("gt", comp_pair[[x]][1],comp_pair[[x]][2]),

tidy=T)

summary(res_sel)

resOrdered <- res_sel[order(res_sel$padj),]

out_file <- paste0("./DEG/",comp_pair[[x]][1],"_vs_",comp_pair[[x]][2],".csv")

write.csv(as.data.frame(resOrdered),out_file,row.names =FALSE,quote = FALSE)

}

rm(list=ls(all=TRUE))

################################################
